# Solenopsin A and analogs exhibit ceramide-like biological activity

**DOI:** 10.1186/s13221-015-0030-2

**Published:** 2015-05-08

**Authors:** Isabella Karlsson, Xin Zhou, Raquela Thomas, Allorie T Smith, Michael Y Bonner, Pooja Bakshi, Ajay K Banga, J Phillip Bowen, Ghassan Qabaja, Shavon L Ford, Matthew D Ballard, Kimberly S Petersen, Xuechen Li, Guangping Chen, Besim Ogretmen, Jin Zhang, E Blake Watkins, Rebecca S Arnold, Jack L Arbiser

**Affiliations:** Department of Dermatology, Emory University School of Medicine, Atlanta, GA USA; Department of Pharmacology and Molecular Sciences, The Johns Hopkins University School of Medicine, Baltimore, MD USA; Department of Biochemistry and Molecular Biology, Medical University of South Carolina, Charleston, SC USA; Department of Pharmaceutical Sciences, School of Pharmacy, Union University, Jackson, TN USA; Department of Physiology and Renal Division, Emory University School of Medicine, Atlanta, GA USA; Center for Drug Design, Department of Pharmaceutical Sciences, College of Pharmacy, Mercer University, Atlanta, GA USA; Department of Chemistry & Biochemistry, University of North Carolina Greensboro, Greensboro, NC USA; Department of Urology, Emory University School of Medicine, Atlanta, GA USA; Atlanta Veterans Administration Hospital, and Winship Cancer Institute, Emory University, Atlanta, GA USA

**Keywords:** Solenopsin A, Ceramide, Akt, Mitophagy, Reactive oxygen

## Abstract

**Background:**

(−)-Solenopsin A is a piperidine alkaloid that is a component of the venom of the fire ant *Solenopsis invicta*. Previously, we have demonstrated that solenopsin exhibit anti-angiogenic activity and downregulate phosphoinositol-3 kinase (PI3K) in the p53 deficient renal cell carcinoma cell line 786-O. Solenopsin has structural similarities to ceramide, a major endogenous regulator of cell signaling and cancer therapy induced apoptosis.

**Methods:**

Different analogs of solenopsin were synthesized in order to explore structure-activity relationships. The anti-proliferative effect of solenopsin and analogs was tested on six different cell lines, including three tumor cell lines, two normal cutaneous cell lines, and one immortalized hyperproliferative cell line. FRET-based reporters were used to study the affect of solenopsin and analogs on Akt activity and PDK1 activation and sucrose density gradient fractionation was performed to examine recruitment of PTEN to membrane rafts. Western-blotting was used to evaluate the affect of solenopsin and analogs on the Akt and the MAPK 44/42 pathways in three different tumor cell lines. Measurement of cellular oxygen consumption rate together with autophagy staining was performed to study mitochondrial function. Finally, the affect of solenopsin and analogs on ROS production was investigated.

**Results:**

In this paper we demonstrate that solenopsin analogs with potent anti-proliferative effects can be synthesized from inexpensive dimethylpyridines. To determine whether solenopsin and analogs act as ceramide analogs, we examined the effect of solenopsin and analogs on two stereotypic sites of ceramide activity, namely at lipid rafts and mitochondria. We found that native solenopsin, (−)-solenopsin A, inhibits functional Akt activity and PDK1 activation in lipid rafts in a similar fashion as ceramide. Both *cis* and *trans* analogs of solenopsin reduce mitochondrial oxygen consumption, increase reactive oxygen, and kill tumor cells with elevated levels of Akt phosphorylation. However, only solenopsin induces mitophagy, like ceramide.

**Conclusions:**

The requirements for ceramide induced mitophagy and inhibition of Akt activity and PDK1 activation in lipid rafts are under strict stereochemical control. The naturally occurring (−)-solenopsin A mimic some of the functions of ceramide and may be therapeutically useful in the treatment of hyperproliferative and malignant disorders of the skin, even in the presence of elevated levels of Akt.

**Electronic supplementary material:**

The online version of this article (doi:10.1186/s13221-015-0030-2) contains supplementary material, which is available to authorized users.

## Background

**Figure 1:**
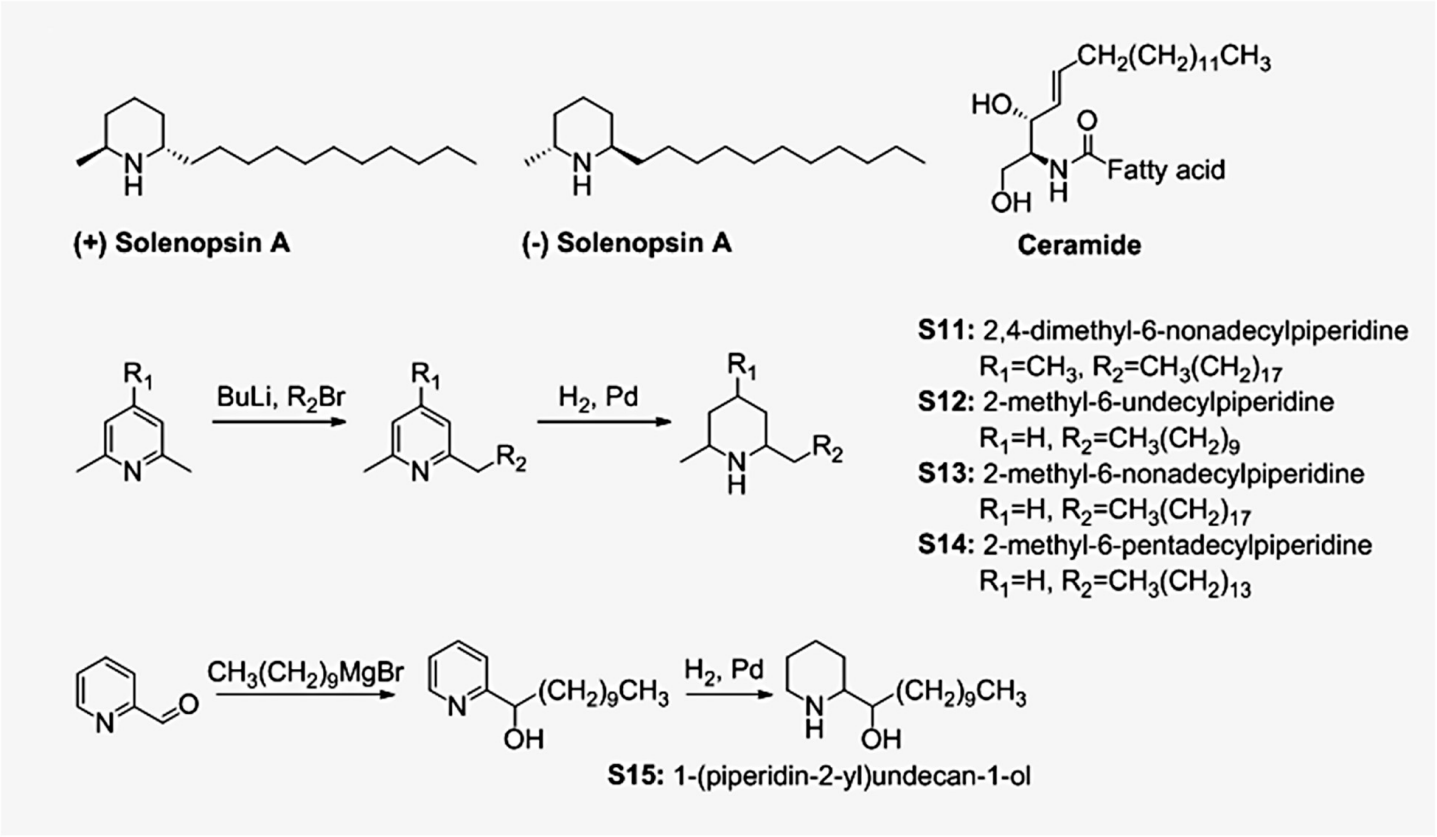
Compounds studied in this paper and synthetic procedures for preparation of compounds S11-S15. (−)-Solenopsin A is a component of the venom of the fire ant solenopsis invicta. (+)-Solenopsin A is the enantiomer of the naturally occurring solenopsin. The structure of solenopsin resembles the structure of ceramides, which are fatty acid amides of sphingosine that play a crucial role in homeostasis of the skin and other organs. Solenopsin analogs **S11**-**S14** were synthesized by deprotonation of 2,6-dimethylpyridine **(S12**-**S14)** or 2,4,6-trimethylpyridine **(S11)** by *n*-butyllithiium, followed by addition of alkyl bromides. Analog **S15** was synthesized by treating pyridine-2-carboxaldehyde with the Grignard reagent decylmagnesium bromide. The solenopsin analogs **(S11**-**S15)** were successfully obtained after hydrogenation of the various 2-alkylpyridines.

(−)-Solenopsin A is a piperidine alkaloid that is a component of the venom of the fire ant *Solenopsis invicta*. Previously, we demonstrated that solenopsin exhibited anti-angiogenic activity and downregulated phosphoinositol-3 kinase (PI3K) in the p53 deficient renal cell carcinoma cell line 786-O [1]. Solenopsin structurally resembles ceramides in chemical structure (Figure 1). Ceramides are fatty acid amides of sphingosine, which play a crucial role in homeostasis of the skin and other organs [2-5]. Defects in the production of ceramides may inhibit physiologic cell death, leading to the persistence of inflammatory and neoplastic cells [5-7]. Large-scale extraction of solenopsin from fire ants is not feasible, and current synthetic routes are multistep routes with low yields. Thus, we devised a simple two-step synthesis of solenopsin analogs starting from inexpensive dimethyl pyridine. We examined the effect of solenopsin and analogs on two stereotypic sites of ceramide activity, lipid rafts and mitochondria [7,8]. We show that native (−)-solenopsin A has ceramide-like activity in lipid rafts and mitochondria. Finally, we show that solenopsin and analogs kill tumor cells regardless of phosphatase and tensin homolog (PTEN) status or Akt activation. Given that loss of PTEN and elevation of Akt are major mechanisms of resistance to chemotherapy [9,10], the use of solenopsin and analogs may be of great utility in treating hyperproliferative skin disorders.

## Methods

### Synthesis

(+)-Solenopsin A and (−)-solenopsin A were synthesized as HCl salts as previously described [1]. Compound **S11**-**S14** were synthesized by deprotonation of 2,6-dimethylpyridine (**S12**-**S14**) or 2,4,6-trimethylpyridine (**S11**) by *n*-butyllithium, followed by addition of alkyl bromides (Figure 1). **S15** was synthesized by treating pyridine-2-carboxaldehyde with the Grignard reagent decylmagnesium bromide (Figure 1). The solenopsin analogs (**S11**-**S15**) were successfully obtained after hydrogenation of the various 2-alkylpyridines (Figure 1). Detailed synthetic procedures and characterization data can be found in the Additional file 1.

### Cells and culture conditions

In this study eight different cell lines were used: human A375 melanoma cells, human A2058 melanoma cells, immortalized murine endothelial SVR cells [11-13], primary human melanocytes, primary human keratinocyts, HaCaTs (immortalized human keratinocytes), murine embryonic NIH3T3 fibroblast cells, and human UM-SCC1A squamous carcinoma cells. All cell lines were grown in DMEM with 10% fetal bovine serum, except for primary keratinocytes which were grown in serum free keratinocyte growth media and primary melanocytes that were grown in complete melanocyte growth media

### Proliferation studies

A375, A2058, SVR, primary melanocyte, primary keratinocyte, and HaCaT cells were treated with test compounds for 24 hours, followed by cell counting with a Coulter Counter. All compounds were tested in quadruplicates. For A375s, A2058s, SVRs, and primary melanocytes 50,000 cells/well were plated. Due to difficulty growing the cells, primary keratinocytes and HaCaTs were plated at a concentration of 20,000 cells/well, and 15,000 cells/well respectively. All cells were treated with 20 μM of ceramide C2. A375s, A2058s, and SVRs were treated with 10 μM of (−)-solenopsin A, (+)-solenopsin A, or analogs **S11**-**S15**, whereas primary melanocytes, primary keratinocytes, and HaCaTs were treated with 20 μM of solenopsin and analogs.

### FRET-based reporter construct

The development of the biocensors Lyn-PARE and AktAR has been described previously [14,15]. Briefly, AktAR was generated by a fluorescent protein pair, cerulean and cpVE172, sandwiching a forkhead-associated binding domain (FHA1) and an Akt substrate domain (FOXO) [15]. Lyn-PARE was generated by sandwiching full-length PDK1 between a FRET pair, ECFP (cyan fluorescent protein) and citrine (yellow fluorescent protein) [16], and a motif generated from the Lyn-kinase gene was added to the 5’-end to target the construct to raft microdomains [14].

### Cell transfection and imaging

Cell transfection and imaging was conducted as previously described [14,15]. NIH3T3 cells were treated for 1 h with DMSO solutions of ceramide C2 (50 μM), (+)-solenopsin A (10 and 20 μM), (−)-solenopsin A (10 and 20 μM), and analogs **S11-S15** (10 μM). A more detailed description of the experimental procedure can be found in the Additional file 1.

### Sucrose density gradient fractionation

Cells were treated for 1 h with 20 μM DMSO solutions of (+)-solenopsin A, (−)-solenopsin A, analogs (**S12-S15**), or 50 μM of ceramide. Lipid raft fractionation was performed with a 5-40% sucrose discontinuous gradient as previously described [17,18]. After ultracentrifugation, thirteen 385 μL fractions were collected, starting from the top of the tube. Equal volumes of each fraction were analyzed by Western blot with rabbit polyclonal antibodies for caveolin-1 and PTEN. A more detailed description of the experimental procedure can be found in the Additional file 1.

### Western blot analysis

Cells were grown in T-25 flasks until 80% confluent followed by treatment for 24 h with 10 μM DMSO solutions of (+)-solenopsin A, (−)-solenopsin A, or analogs (**S11-S15**). Sample aliquots normalized for protein quantities were size fractionated by 10% SDS-PAGE, and the proteins were transferred to a PVDF membrane. The blots were incubated in blocking solution; TBS with 5% (wt/vol) powdered nonfat milk for 1 h at room temperature, followed by incubation over night with rabbit polyclonal p-Akt S473, p-MAPK 44/42, and Β-actin.

### Measurement of oxygen consumption rate (OCR)

UM-SCC1A cells were plated 15,000 cells/well in 200 μl DMEM supplemented with 10% FBS and 1% penicillin-streptomycin in each well of a 96-well Seahorse plate and incubated overnight at 37°C with 5% CO_2_. Cells were treated with 10 μM of compound or DMSO and incubated for 24 hours. OCR was measured as pmoles O_2_/minute using the Seahorse Biosciences instrument per manufacturer’s instructions. Protein amounts in each well were quantified using the Thermo Scientific Pierce Protein Assay, per manufacturer’s instructions.

### Autophagosome staining

UM-SCC1A cells were treated with DMSO (control) or 10 μM of (−)-solenopsin A or analog **S14** for 18 h. The cells were stained using a Cyto-ID Autophagy Staining assay from Life Technologies.

### Measurement of ROS with dihydroethidium (DHE)

Briefly, A375 and SVR cells were treated for 24 h with 10 μM DMSO solutions of (+)-solenopsin A, (−)-solenopsin A, or analogs (**S11-S15**). Cells were washed, pelleted, suspended in 10 μM DHE and incubated for 10 min. Thereafter cells were counted using a Becton Dickinson FACScan flow cytometer. Mean values of DHE fluorescence intensity were compared and all samples were repeated in triplicate. A more detailed description of the experimental procedure can be found in the Additional file 1.

### Determination of cytotoxicity by MTT assay

MTT reagent was added to the tissue inserts after 72 hours treatment with 10 μM of Solenopsin A and analogs **S12** and **S14** followed by incubation for 3 hours at 37°C with 5% CO_2_. Thereafter, MTT was extracted from the tissues and the absorbance was measured at 570 nm. Cell viability was calculated using a spreadsheet provided by MatTek; viability of less than 50% was determined to be irritant and cytotoxic. H&E staining of the tissues from the cultures inserts were also performed; see Additional file 1 for more detailed experimental procedures.

## Results

### Structure-activity relationships of solenopsin and analogs

**Figure 2:**
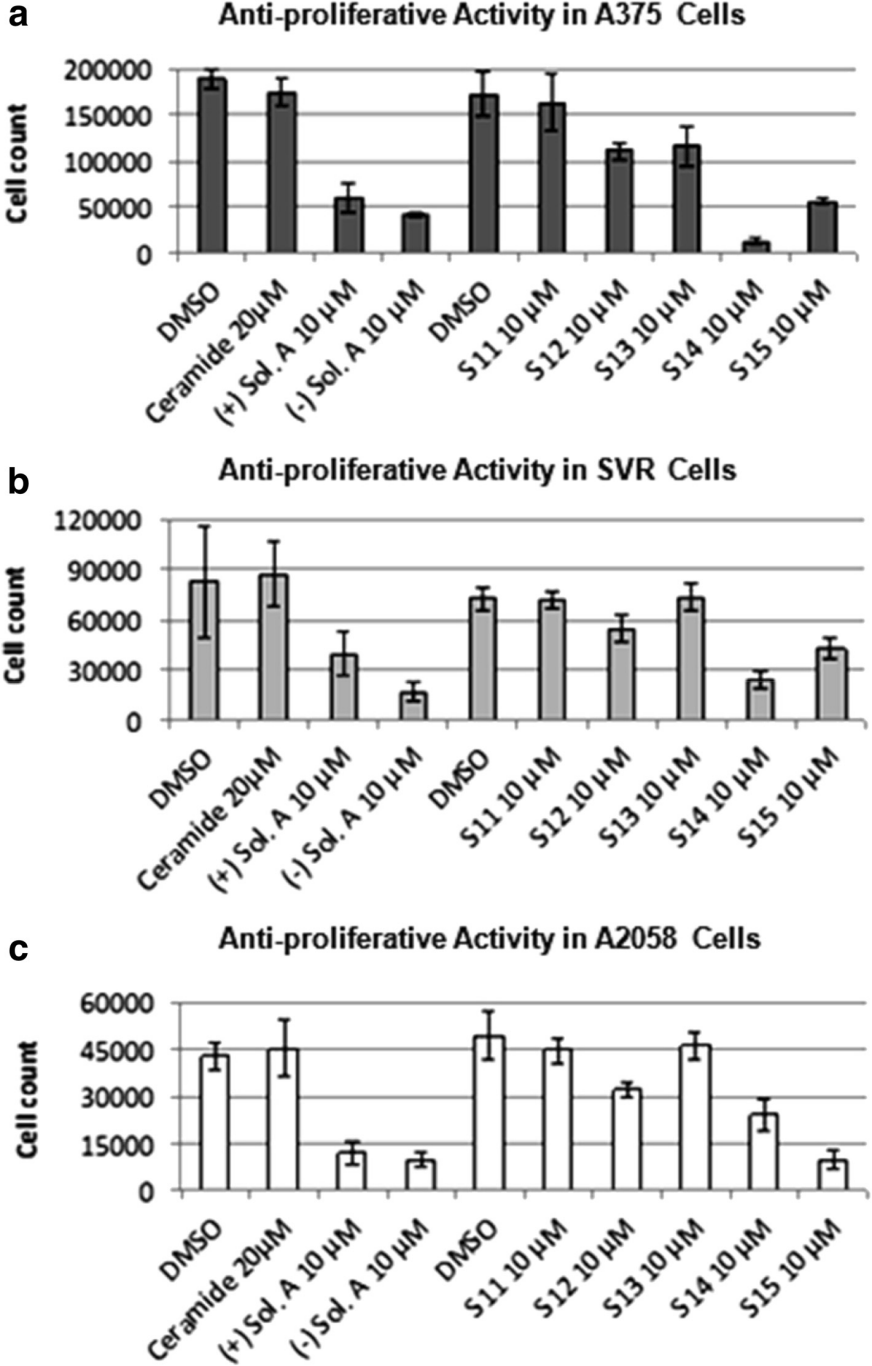
Assessment of anti-proliferative activity for solenopsin and analogs S11-S15. The anti-proliferative effect of (+)-solenopsin A ((+) Sol. A), (−)-solenopsin A ((−) Sol. A), ceramide C2, and solenopsin analogs **S11**-**S15** were evaluated in **(a)** A375 cells, **(b)** SVR cells, and **(c)** A2058 cells. 50,000 cells/well were plated and treated for 24 h with each compound. The first DMSO bar in each chart serves as control for (+)-solenopsin A, (−)-solenopsin A, and ceramide C2. The second DMSO bar is the control for **S11**-**S15**. The displayed data are an average of four experiments ± s.d.

Different analogs of solenopsin were synthesized (Figure 1) in order to explore structure-activity relationships. Three different tumor cell lines, relevant for skin, were used to assess anti-proliferative potency: human A375 melanoma cells, human A2058 melanoma cells, and murine SVR angiosarcoma cells. No significant difference between the naturally occurring compound, (−)-solenopsin A, and its enantiomer (+)-solenopsin A could be seen in any of the tumor cell lines (Figure 2). Both of these compounds have *trans* geometry. The mixture of the two *cis* isomers of solenopsin, **S12**, show weaker anti-proliferative activity than (−)-solenopsin A in all three cell lines (Figure 2), suggesting that the *trans* isomers are more potent than the corresponding *cis* isomers. Elongation of the aliphatic side chain with 8 carbons had a negative effect on potency, as analogs **S11** and **S13** displayed a lower anti-proliferative effect than both solenopsin and **S12**. Also, the only compound that showed no anti-proliferative effect in any of the cell lines was **S11**, which in addition to having the longest aliphatic side chain also has an extra methyl group on the piperidine ring. Interestingly, analog **S14**, which has a 4 carbon longer aliphatic side chain than solenopsin, turned out to be the most potent analog in human melanoma A375 cells and murine angiosarcoma SVR cells (Figure 2a and b). As **S14** was significantly more potent than **S12** in all three cell lines, it would appear as if a 15-carbon side chain is the optimum length for anti-proliferative activity. **S15**, which has the same length side chain as solenopsin but lacks the methyl group and has an extra hydroxyl group, also displayed potent anti-proliferative effect in all three cell lines and in human melanoma A2058 cells it was the most potent analog.

The effect of ceramide, solenopsin A, and analogs **S11**-**S15** was also assessed on two normal cutaneous cell lines, namely primary melanocytes and primary keratinocytes (Additional file 1: Figure S1). In addition, the activity of the compounds was assessed in HaCaTs, which are immortalized hyperproliferative human keratinocytes (Additional file 1: Figure S1). The analogs **S11** and **S13**, which were inactive in the tumor cell lines, did not have any activity in these cell lines either. Ceramide only showed activity in primary melanocytes and keratinocytes (Additional file 1: Figure S1), but not in malignant A375s, A2058s, and SVRs, (Figure 2). Interestingly, HaCat cells, which represent premalignant keratinocytes, are resistant to ceramide, supporting our hypothesis that loss of response to ceramide may represent an early event in skin carcinogenesis. Solenopsin A and the analogs **S12**, **S14**, and **S15** had significant activity in all cell lines, including malignant and primary cell lines (Figure 2 and Additional file 1: Figure S1). Solenopsin A and active analogs were shown to be non-toxic to reconstituted skin equivalents (Additional file 1: Figure S2). Normal keratinization was preserved as assessed by routine histology (data not show).

### Solenopsin inhibits functional Akt activity and PDK1 activation

**Figure 3:**
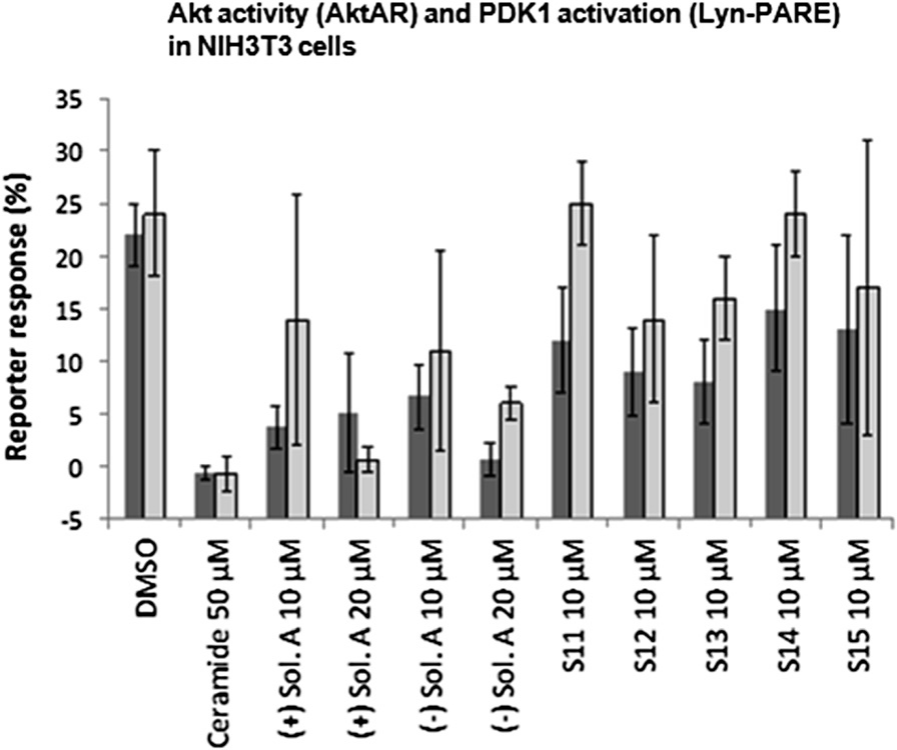
Solenopsin A inhibits Akt activity (AktAR) and PDK1 activation (Lyn-PARE). NIH3T3 cells were transfected with AktAR or Lyn-PARE and serum starved for 24 h, followed by 1 h treatments with DMSO, ceramide C2, (+)-solenopsin A ((+) Sol. A), (−)-solenopsin A ((−) Sol. A) or solenopsin analogs **S11**-**S15**. To study Akt activity and PDK1 activation, 50 ng/mL of PDGF was added followed by immediate imaging. FRET ratio of regions of interest at cell cytosol and at cell periphery representing the plasma membrane were used, respectively. Akt activity is measured by AktAR response (dark grey columns) and PDK1 activation by Lyn-PARE response (light grey columns). All the ratios were normalized with the ratio before PDGF addition. Data shown are an average of at least three experiments ± s.d.

Ceramides are found in the cell membrane where they act as signaling molecules and play a role in a variety of physiological conditions, such as: differentiation, proliferation, programmed cell death, apoptosis etc. [19]. The underlying mechanisms are complex and the exact means by which ceramides function as signaling molecules are not clear. However, there are a number of studies that show that ceramide inhibit the PI3K/Akt pathway [14]. To determine whether solenopsin A and analogs have similar modes of action as ceramide, we employed a FRET-based Akt activity reporter (AktAR) and a PDK1 activation reporter targeted to membrane rafts (Lyn-PARE) [14,15]. The AktAR construct contains a binding domain (FHA1), a substrate domain (FOXO), and two fluorescent proteins that constitute a FRET pair. This reporter functions as surrogate substrate for Akt and phosphorylation of the substrate leads to a detectable change in FRET [15]. Lyn-PARE contains the full-length PDK1 protein flanked by two fluorescent proteins that constitute a FRET pair. This construct also contains a motif derived from Lyn-kinase, which targets it to membrane rafts. Activation of PDK1 leads to a conformational change and thereby a detectable change in FRET [14]. Previously, these reporters have been used to show that ceramide treatment inhibits PDGF-induced Akt activity and activation of PDK1 in membrane rafts [14]. At 20 μM concentrations both (+)- and (−)-solenopsin A inhibited Akt activity (AktAR) and PDK1 activation (Lyn-PARE) to similar extent as treatment with 50 μM of ceramide (Figure 3). At 10 μM concentrations some inhibition of Akt activity and PDK1 activation could be seen for both (+)- and (−)-solenopsin A. At 10 μM **S13** and possibly also **S12** displayed some inhibition of Akt activity and PDK1 activation. No significant inhibition could be seen for any of the other solenopsin analogs (Figure 3).

### Translocation of PTEN to membrane rafts

To examine whether solenopsin recruits PTEN to membrane rafts, as ceramide does, sucrose density gradient fractionation was performed followed by evaluation of the PTEN levels in each fraction. PTEN negatively regulates the PI3K/Akt signaling, which takes place in the lipid raft regions, by converting PIP_3_ (phosphatidylinostol (3,4,5)-triphosphate) to PIP_2_ (phosphatidylinositol (4,5)-biphosphate). As most PTEN is usually localized to nonraft regions [14,20,21], studies suggest that ceramide inhibits PI3K/Akt signaling by translocating PTEN from nonraft regions into lipid rafts [15,22,23].

As expected, the amounts of PTEN seem to be higher in lipid raft fractions from cells treated with ceramide (fraction 1–4) (Additional file 1: Figure S3). The compound that appeared to have the largest amount of PTEN in the raft fractions was (−)-solenopsin A (Additional file 1: Figure S3). (+)-solenopsin A and **S12**-treated cells showed similar amounts of PTEN in the raft fractions as the ceramide treated cells (Additional file 1: Figure S3). The rest of the analogs (**S13**-**S15**) appeared to have similar or even lower amounts of PTEN in lipid rafts than the control (Additional file 1: Figure S3). It is worth pointing out that for these experiments the use of a loading control is not possible. Therefore, these results should only be regarded as an indication of the compounds ability to recruit PTEN to membrane rafts. However, the FRET-based analysis was largely consistent with the PTEN localization as demonstrated by sucrose density fractionation.

### Solenopsin A and analogs effect on signaling pathways is context dependent

A375 (human melanoma), SVR (murine angiosarcoma), and A2058 (human melanoma) cells treated with solenopsin A and analogs were evaluated by Western-blotting with p-Akt S473, p-MAPK 44/42, and B-actin (Additional file 1: Figure S4 and Additional file 1: Table S1). In A375 human melanoma cells, an up-regulation of p-Akt S473 and p-MAPK 44/42 could be seen for (+)- and (−)-solenopsin A, as well as for analogs **S12**-**S15**. In SVR murine angiosarcoma cells on the other hand, p-Akt S473 and p-MAPK 44/42 were down-regulated in all treatment groups, and especially in cells treated with analog **S15**. The results for the human melanoma A2058 cells were similar to A375 cells but not as pronounced, i.e. there is a slight up-regulation of p-Akt S473 and p-MAPK 44/42 in some of the treatment groups compared to the control. Both human melanoma cell lines (A375 and A2058) have intact p53 and loss of p16ink4a [24], whereas the murine angiosarcoma cell line (SVR) has defective p53 function due to the presence of SV40 large T antigen [11]. This may account for the observed difference in cell signaling between these cell lines.

### Solenopsin A and analogs affect mitochondrial function

**Figure 4:**
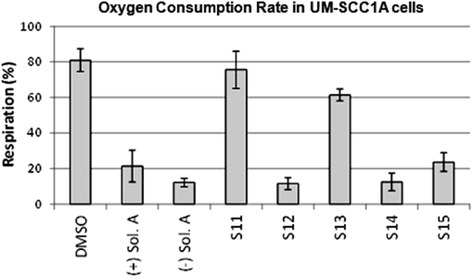
Solenopsin A and analogs reduce oxygen consumption rate. UM-SCC1A cells were plated 15,000/well and treated for 24 h with 10 μM of (+)-solenopsin A ((+) Sol. A), (−)-solenopsin A ((−) Sol. A), and solenopsin analogs **S11**-**S15**. Oxygen consumption rate (OCR) was measured as pmoles O_2_/minute using a Seahorse Biosciences instrument. Data shown are an average of three experiments ± s.d.

Although solenopsin A recruits PTEN to lipid rafts (Additional file 1: Figure S3), it does not appear to be enough to dephosphorylate Akt (Additional file 1: Figure S4) in the human melanoma cell lines A375 and A2058 (both are wild type p53) like it did in 786-O renal cell carcinoma cells (mutant p53) [1]. One explanation for the anti-proliferative activity of solenopsin and analogs could be that they, like ceramide, also localize to mitochondria and increase the production of reactive oxygen [25], thereby causing mitophagy and cell death. Cellular oxygen consumption rate (OCR) can be indicative of mitochondrial function. To investigate if solenopsin A and analogs alter mitochondrial function in human head and neck squamous carcinoma cells like ceramide [8], UM-SCC1A cells were treated with 10 μM of solenopsin and analogs, followed by incubation for 24 hours. Cells treated with (+)- and (−)-solenopsin A, **S12**, **S14**, and **S15** displayed reduced OCR compared to control, while cells treated with **S13** have slightly reduced OCR and cells treated with **S11** show little change in OCR compared to the control (Figure 4). To verify that mitophagy is the reason for the observed decrease in OCR, a Cyto-ID Autophagy Staining assay was used. The dye stains autophagosomes and co-localizes with LC3-II. UM-SCC1A cells were treated with DMSO (control) and 10 μM of (−)-solenopsin A or analog **S14** for 18 h. A significant increase in fluorescence could be seen for (−)-solenopsin A compared to the control, which verifies that (−)-solenopsin A induces autophagy (Additional file 1: Figure S5). Analog **S14**, on the other hand, does not appear to induce autophagy; instead it looks as if **S14** may even decrease basal levels of autophagy (Additional file 1: Figure S5).

### Solenopsin A and analogs elevate ROS levels

**Figure 5:**
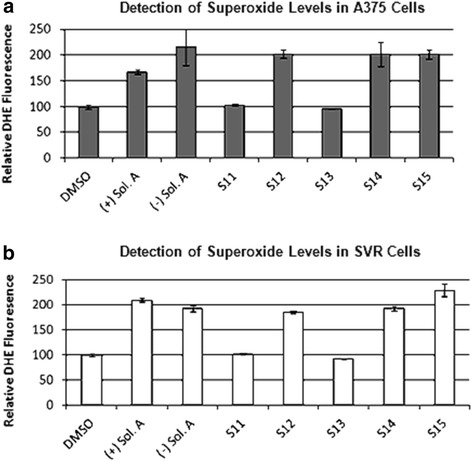
Solenopsin A and analogs increase superoxide levels. Investigation of solenopsin and analogs **S11**-**S14** effect on superoxide levels in **(a)** A375 cells, and **(b)** SVR cells. Cells were treated for 24 h with 10 μM of (+)-solenopsin A ((+) Sol. A), (−)-solenopsin A ((−) Sol. A), and solenopsin analogs **S11**-**S15**. Cells were trypsined and incubated for 10 min in 10 μM dihydroethedium, followed by analysis with a FACScan flow cytometer. Data shown are an average of three experiments ± s.d.

Ceramide is known to increase ROS production [25-27] and given that Akt phosphorylation was elevated by solenopsin and analog treatment in human melanoma cells, and that elevated phosphorylation of Akt is a common response to superoxide, we examined levels of superoxide. There was a marked increase in superoxide levels, as measured by DHE fluorescence, in both A375 human melanoma and SVR murine angiosarcoma cells treated with the compounds (+)- and (−)-solenopsin A, **S12**, **S14**, and **S15** ranging from 1.7-2.3 fold compared to vehicle treated cells (Figure 5). Compounds **S11**, and **S13** had no effect on superoxide levels (Figure 5).

## Discussion

Ceramides play a role in physiologic cell death, as they are involved in removal of undesired cells, and thus limit both inflammation and neoplasia [28,29]. Ceramides have also been implicated in mediating cell death due to chemotherapy and radiation, and inability to generate ceramides is linked to resistance to these treatments [7,28-30]. Thus, restoration of ceramide is a potential anti-angiogenic and anti-inflammatory modality, but is complicated by difficult synthesis, low stability and rapid metabolism. Therefore, analogs that do not suffer from these disadvantages could be therapeutically beneficial. We noted the similarity of solenopsin to ceramide and hypothesized that solenopsin and analogs might act as ceramide-like agonists. We thus evaluated their ceramide-like activity at both the lipid membrane and mitochondria.

A major obstacle to the widespread use of solenopsin is obtaining sufficient quantities for preclinical and clinical studies. Extraction of solenopsin from ants is not feasible, and therefore large scale synthesis is required. All current synthetic methods suffer from reliance on expensive reagents and multiple steps [31-33]. In this paper, we demonstrate that solenopsin analogs can be synthesized by lithiation of inexpensive industrial dimethylpyridines, followed by alkylation of the lithiated pyridines with alkyl halides, which can be varied. Finally, the alkylated pyridine is hydrogenated to give the solenopsin analogs (Figure 1). Each of these steps is amenable to scale up for industrial production [32,34]. In a previous study by our group we showed that solenopsin analogs with shorter aliphatic side chains lacked anti-proliferative activity in murine angiosarcoma SVR cells [1]. Here we demonstrate that solenopsin analogs with 8 carbon longer side chains (**S11** and **S13**) also lack significant anti-proliferative activity in murine SVR angiosarcoma and human melanoma (A375 and A2058) cells. On the other hand, **S14**, which has a 4 carbon longer side chain, and **S15**, which has the same length side-chain but contains a hydroxyl group and lacks the methyl group, are equally as potent as (−)-solenopsin A in all three cell lines. Thus, optimal length of the aliphatic side chain appears to be associated with high anti-proliferative activity.

**Figure 6:**
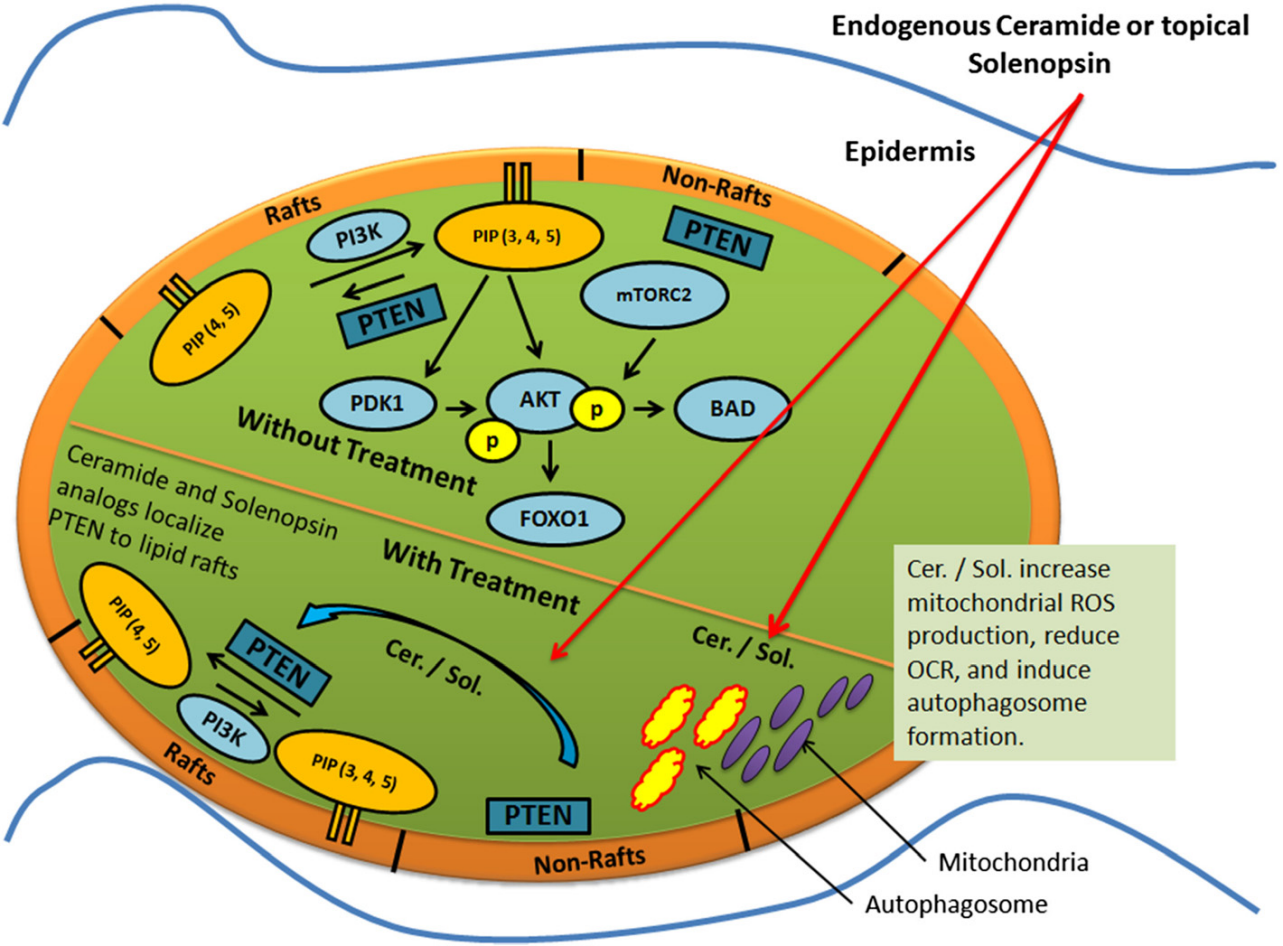
A model for the effect of Ceramide and Solenopsin on cell function. Ceramide (Cer.) and solenopsin (Sol.) derivatives inhibit the AKT pathway by translocating PTEN from non-raft regions into the rafts. Ceramide and solenopsin also increase mitochondrial ROS production, reduce oxygen consumption (OCR), and induces autophagosome formation.

One of the modes of ceramide’s activity is downregulation of Akt, a serine-threonine kinase that plays a central role in protecting tumor cells from apoptosis [35,36]. Akt, which originally was discovered as a viral oncogene, has transforming activity in multiple cell types. PTEN is a tumor suppressor that inhibits Akt activation in lipid rafts. We used A375 human melanoma cells, which express wild type PTEN [37], to assess the effect of solenopsin A on PTEN localization. Indeed, solenopsin A did appear to cause relocalization of PTEN to lipid rafts (Additional file 1: Figure S3). FRET-based reporters were used to show that solenopsin A inhibits Akt activity (AktAR) and PDK1 activation (Lyn-PARE) in lipid rafts to the same extent as ceramide in NIH3T3 murine embryonic fibroblast cells (Figure 3). Unexpectedly, we found that solenopsin A increases Akt phosphorylation in cells with wild type p53 (A375 and A2058), while it decreases Akt activation in cells with defective p53 function (SVR and 786-O) (Additional file 1: Figure S4), thus demonstrating a context dependent effect on tumor cells [1,38]. Taken together it appears as if relocalization of PTEN to lipid rafts is insufficient to block Akt activation, at least in cells with wild type p53. In addition, solenopsin A is highly effective in killing A375 human melanoma cells, despite no reduction of Akt phosphorylation. This implies that solenopsin’s killing of tumor cells with wild type PTEN may not depend exclusively on Akt deactivation, but may involve additional events, such as mitochondrial induced cell death. Indeed, investigation of solenopsin and analogs affect on OCR-levels revealed a similar trend as the proliferation assays. Solenopsin A and the analogs **S12**, **S14** and **S15** all markedly decreased the OCR levels (Figure 4) in UM-SCC1A cells. Interestingly, autophagy staining showed that (−)-solenopsin A (*trans* geometry) but not **S14** (*cis* geometry) induced mitophagy (Additional file 1: Figure S5), indicating strict stereochemical requirements for induction of mitophagy. Thus, the *trans* geometry is not essential for anti-proliferative activity, although it appears to be required for ceramide-like mitophagy activity. Further, our study of superoxide levels displayed a similar pattern as the proliferation and OCR assays, i.e. solenopsin A, **S12**, **S14** and **S15** substantially increased superoxide, whereas **S11** and **S13** showed no effect (Figure 5). Figure 6 is a suggested model, based on our results, which show the effect of ceramide and solenopsin on cell function. The FDA requires all new topical and transdermal products to be evaluated for skin irritation and sensitization [39]. A 3D cell culture of human skin keratinocytes has been evaluated by European Center for the Validation of Alternative Methods as an alternative to the rabbit draize test, to avoid animal usage [40]. In our study, solenopsin and analogs **S12**, **S14** were found to be non-irritant (Additional file 1: Figure S2). Thus, solenopsin and analogs have the potential for further development for topical treatment of both tumors and inflammatory disorders that are deficient in ceramide.

## Conclusions

In this work we show that the naturally occurring (−)-solenopsin A exhibit ceramide-like biological activity in human melanoma cell lines and could therefore be useful in treating hyperproliferative skin disorders. First, we demonstrate that native (−)-solenopsin A mimics canonical functions of ceramide on cells, namely inhibition of Akt activity and PDK1 activation in lipid rafts as well as induction of mitophagy (Figure 6). Differences in *cis-trans* geometry and chain length abrogate these functions. Second, the response to solenopsin differs in cells of various genetic backgrounds, with no effect or elevation of Akt phosphorylation in cells with wild type p53 (A375, A2058), while decreasing Akt phosphorylation in cells with defective p53 (SVR, 786-O). Solenopsin and analogs are also effective in killing cells with elevated Akt phosphorylation, which is an adverse prognostic factor in most cancers. Based upon the novel hypothesis that restoration of ceramide-like signaling may be beneficial in the treatment of neoplastic and hyperproliferative skin disorders, our assessment of solenopsin and analogs on normal cutaneous cells and premalignant cells demonstrate the potential of solenopsin and analogs to treat hyperproliferative disorders when compared to ceramide.

### Supporting data

The data set supporting the results of this article is included within the article and its additional file: Supplementary Material Solenopsin.

## Additional file

Additional file 1: **Supplementary Material Solenopsin.**

## Abbreviations

DHE: Dihydroethidium
FOXO: Forkhead box protein
OCR: Oxygen consumption rate
PI3K: Phosphoinositol-3 kinase
PIP_2_: Phosphatidylinositol (4,5)-biphosphate
PIP_3_: Phosphatidylinostol (3,4,5)-triphosphate
PTEN: Phosphatase and tensin homolog
