# Supplementary material for: Solenopsin A and analogs exhibit ceramide-like biological activity
Source: Vasc Cell. 2015 May 8;7:5. doi: 10.1186/s13221-015-0030-2 (PMC4443652; doi:10.1186/s13221-015-0030-2)
Supplement: Supplementary file 1 — Supplementary Material Solenopsin. [file 13221_2015_30_MOESM1_ESM.pdf]

Supplementary Material for:

Solenopsin A and Analogs Exhibit Ceramide-Like Biological Activity

Isabella Karlsson[1], Xin Zhou[2], Raquela Thomas[3], Allorie T. Smith[4], Michael Y. Bonner[1], Pooja Bakshi[6], Ajay K. Banga[6], J. Phillip Bowen[6], Ghassan Qabaja[7], Shavon L. Ford[7], Matthew D. Ballard[7], Kimberly S. Petersen[7], Xuechen Li[5], Guangping Chen[5], Besim Ogretmen[3], Jin Zhang[2], E. Blake Watkins[4], Rebecca S. Arnold[8], Jack L. Arbiser[1, 9]

[1] Department of Dermatology, Emory University School of Medicine, Atlanta, GA.
[2] Department of Pharmacology and Molecular Sciences, The Johns Hopkins University School of Medicine, Baltimore, MD.
[3] Department of Biochemistry and Molecular Biology, Medical University of South Carolina, Charleston, SC.
[4] Department of Pharmaceutical Sciences, School of Pharmacy, Union University, Jackson, TN.
[5] Department of Physiology and Renal Division, Emory University School of Medicine, Atlanta, GA.
[6] Center for Drug Design, Department of Pharmaceutical Sciences, College of Pharmacy, Mercer University, Atlanta, GA.
[7] Department of Chemistry & Biochemistry, University of North Carolina Greensboro, Greensboro, NC.
[8] Department of Urology, Emory University School of Medicine, Atlanta, GA
[9] Atlanta Veterans Administration Hospital, and Winship Cancer Institute, Emory University Atlanta, GA.

Corresponding author:
Jack L. Arbiser
Department of Dermatology, Emory University School of Medicine
WMB 5309, 1639 Pierce Drive
Atlanta, GA 30322
Tel (404) 727-5063
Fax (404) 727-5897
Email: jarbise@emory.edu

Table of Contents

TABLE OF CONTENTS 2

SYNTHETIC PROCEDURES 3

CHEMICALS 3
GENERAL PROCEDURE FOR PREPARATION OF SOLENOPSIN ANALOGS 3
S11: 2,4-DIMETHYL-6-NONADECYLPIPERIDINE 3
S12: 2-METHYL-6-UNDECYLPIPERIDINE 4
S13: 2-METHYL-6-NONADECYLPIPERIDINE 4
S14: 2-METHYL-6-PENTADECYLPIPERIDINE 5
S15: 1-(PIPERIDIN-2-YL)UNDECAN-1-OL 5

ASSESSMENT OF ANTI-PROLIFERATIVE ACTIVITY FOR CERAMIDE, SOLENOPSIN A, AND ANALOGS S11-S15 6

FIGURE S1 | ASSESSMENT OF ANTI-PROLIFERATIVE ACTIVITY FOR CERAMIDE, SOLENOPSIN A, AND ANALOGS S11-S15. 7

AKT ACTIVITY AND PDK1 ACTIVATION - CELL TRANSFECTION AND IMAGING 8

DETERMINATION OF CYTOTOXIC CAPACITY OF SOLENOPSIN A AND ANALOGS S12 AND S14 9

DETERMINATION OF CYTOTOXICITY BY MTT ASSAY 9
FIGURE S2 | DETERMINATION OF CELL VIABILITY AND SKIN IRRITATION BY USING MTT ASSAY 9
H&E STAINING. 10

TRANSLOCATION OF PTEN TO MEMBRANE RAFTS 11

SUCROSE DENSITY GRADIENT FRACTIONATION 11
FIGURE S3 | LIPID RAFT FRACTIONATION 12

SOLENOPSIN A AND ANALOGS EFFECT ON SIGNALING PATHWAYS 13

FIGURE S4 | THE EFFECT OF SOLENOPSIN A AND ANALOGS ON THE EXPRESSION OF P-AKT AND P-PMAPK 44/42. 13
TABLE S1 | QUANTIFICATION OF WESTERN BLOTS 14

AUTOPHAGOSOME STAINING TO STUDY MITOCHONDRIAL FUNCTION 15

FIGURE S5 | AUTOPHAGY STAINING CONFIRMS THAT SOLENOPSIN A INDUCES AUTOPHAGY 15

MEASUREMENT OF ROS WITH DIHYDROETHIDIUM (DHE) 16

REFERENCES 17

Synthetic Procedures

Chemicals

Unless otherwise indicated, reagents were obtained from commercial suppliers and used without further purification.

General Procedure for Preparation of Solenopsin Analogs

2,6-Dimethyl pyridine or 2,4,6-trimethyl pyridine (1.35 equiv.) was added dropwise to a stirred solution of n-BuLi (2M, 1.5 equiv.) in cyclohexane at 0 °C. After 30 min of stirring at 0 °C, the alkylbromide (1.0 equiv) was added dropwise and the reaction mixture was allowed to reach room temperature. The slurry was stirred at room temperature for another 4h, followed by addition of ice water. The obtained water-mixture was extracted three times with ethyl acetate. The organic layers were combined and washed with brine, dried over $MgSO_4$, filtered, and concentrated under reduced pressure. The crude compounds were purified by flash chromatography on silica gel. The resulting substituted pyridines (1.0 g) were reduced to the corresponding piperidines through catalytic hydrogenation at 50 psi for 12 hours in the presence of palladium (10 mol%) and rhodium on carbon (10 mol%) in absolute ethanol (80 mL). The mixture was filtered through Celite and concentrated under vacuum. The residue was passed through a short pad of silica, eluting with 20% (10% NH_4OH:MeOH) in ethyl acetate, to give the products after concentration. ^{1}H NMR of compounds **S11**-**S14** showed the presence of a single diastereomer. Based on the work of Pianaro *et al*, we expect the diastereomer to be the *cis* isomer (Pianaro *et al.*, 2012).

S11: 2,4-Dimethyl-6-nonadecylpiperidine

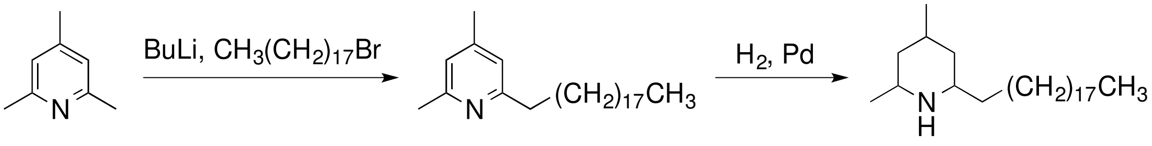

S11 precursor **S11**

Compound **S11** was prepared from 2.5 mL of 2,4,6-trimethyl pyridine (19 mmol) and 4.8 mL of 1-bromooctadecane (14 mmol) according to the general procedure. The crude 2,4-dimethyl-6-nonadecylpyridine was purified by flash chromatography (silica: ethyl acetate/hexanes 1:19), which afforded 2,4-dimethyl-6-nonadecylpyridine, the **S11** precursor, as an off-white solid (3.71g, 71% yield). ^{1}H NMR ($CDCl_3$) δ: 6.76 (d, 2H, *J*=6.4), 2.67 (t, 2H, *J*=8.0), 2.46 (s, 3H), 2.25 (s, 3H), 1.70–1.57 (m, 2H), 1.37–1.15 (m, 32H), 0.86 (t, 3H, *J*=7.0). UPLC-MS (ESI): 374.4 ($M+H^+$).

Hydrogenation according to the general procedure gave the product (**S11**) as a white solid (893 mg, 88%). mp 56.3-57.2 °C. ^{1}H NMR ($CDCl_3$) δ: 2.69-2.64 (m, 1H), 2.54-2.48 (m, 1H), 1.66-1.58 (m, 2H), 1.52-1.42 (m, 1H), 1.40-1.25 (m, 36), 1.09 (d, 3H, *J*=6.0), 0.89 (m, 6H), 0.75-0.64 (m, 2H). UPLC-MS (ESI): 380.4 ($M+H^+$).

S12: 2-Methyl-6-undecylpiperidine

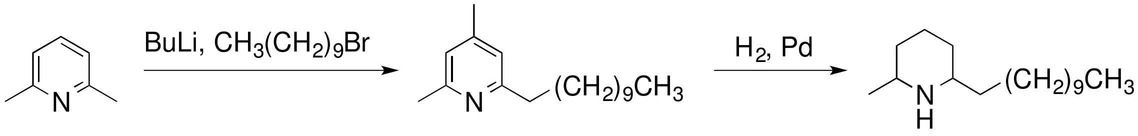

Compound **S12** was prepared from 3.0 mL of 2,6- dimethyl pyridine (26 mmol) and 4.0 mL of 1-bromodecane (19 mmol) according to the general procedure. The crude 2-methyl-6-undecylpyridine was purified by flash chromatography (silica: ethyl acetate/hexanes 1:9), which afforded 2-methyl-6-undecylpyridine, the **S12** precursor, as a yellow oil (2.63 g, 60% yield). ^{1}H NMR ($CDCl_3$) δ: 7.47 (t, 1H, *J*=7.6), 6.96–6.93 (m, 2H), 2.74 (t, 2H, *J*=8.0), 2.52 (s, 3H), 1.73–1.67 (m, 2H), 1.37–1.25 (m, 18H), 0.88 (t, 3H, *J*=7.0). UPLC-MS (ESI): 248.2 ($M+H^+$).

Hydrogenation according to the general procedure gave the product (**S12**) as a clear oil which solidified upon cooling (832 mg, 81%). ^{1}H NMR ($CDCl_3$) δ: 2.66-2.61 (m, 1H), 2.51-2.45 (m, 1H), 1.79-1.74 (m, 1H), 1.66-1.57 (m, 2H), 1.35-1.25 (m, 21H), 1.09-1.08 (d, 3H, *J*=4.4), 1.06-0.98 (m, 2H), 0.88 (t, 3H, *J*=7.2). UPLC-MS (ESI): 254.1 ($M+H^+$). ^{1}H NMR and ^{13}C NMR were according to the literature.(H. M. T. Bandara Herath and Nanayakkara, 2008)

S13: 2-Methyl-6-nonadecylpiperidine

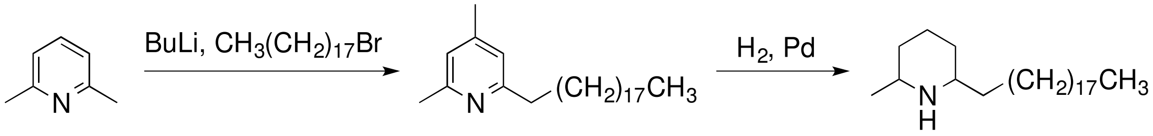

Compound **S13** was prepared from 2.2 mL of 2,6-dimethyl pyridine (19 mmol) and 4.8 mL of 1-bromooctadecane (14 mmol) according to the general procedure. The crude 2-methyl-6-nonadecylpyridine was purified by flash chromatography (silica: ethyl acetate/hexanes 1:9), which afforded 2-methyl-6-nonadecylpyridine, the **S13** precursor, as an off-white solid (4.57g, 91% yield). ^{1}H NMR ($CDCl_3$) δ: 7.45 (t, 1H, *J*=7.6), 6.94–6.91 (m, 2H), 2.72 (t, 2H, *J*=8.0), 2.51 (s, 3H), 1.70–1.64 (m, 2H), 1.37–1.15 (m, 32H), 0.88 (t, 3H, *J*=6.8). UPLC-MS (ESI): 360.4 ($M+H^+$).

Hydrogenation according to the general procedure gave the product (**S13**) as a white solid (859 mg, 84%). mp 46.5-47.3 ^{o}C. ^{1}H NMR ($CDCl_3$) δ: 2.65-2.62 (m, 1H), 2.50-2.48 (m, 1H), 1.78-1.74 (m, 1H), 1.66-1.58 (m, 2H), 1.37-1.25 (m, 37H), 1.08-1.07 (d, 3H, *J*=4.4), 1.05-0.95 (m, 2H), 0.88 (t, 3H, *J*=6.4). UPLC-MS (ESI): 366.3 ($M+H^+$).

S14: 2-Methyl-6-pentadecylpiperidine

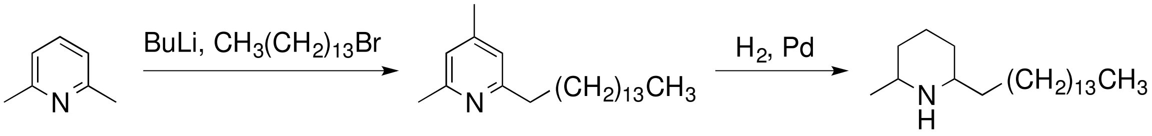

S14 precursor **S14**

Compound **S14** was prepared from 2.2 mL of 2,6-dimethyl pyridine (19 mmol) and 4.1 mL of 1-bromopentadecane (14 mmol) according to the general procedure. The crude 2-methyl-6-pentadecylpyridine was purified by flash chromatography (silica: ethyl acetate/hexanes 1:20), which afforded 2-methyl-6-pentadecylpyridine, the **S14** precursor, as a clear oil (4.2 g, 99%). ^{1}H NMR ($CDCl_3$) δ: 7.45 (t, 1H, *J* 7.6), 6.94–6.91 (m, 2H), 2.72 (t, 2H, *J*=8.0), 2.51 (s, 3H), 1.70–1.62 (m, 2H), 1.35–1.19 (m, 24H), 0.88 (t, 3H, *J*=6.8). UPLC-MS (ESI): 304.2 ($M+H^+$).

Hydrogenation according to the general procedure gave the product (**S14**) as a clear oil (839 mg, 82%). ^{1}H NMR ($CDCl_3$) δ: 2.62-2.61 (m, 1H), 2.48-2.46 (m, 1H), 1.77-1.74 (m, 1H), 1.64-1.57 (m, 2H), 1.35-1.25 (m, 29H), 1.06-1.05 (d, 3H, *J*=6.0), 1.04-0.95 (m, 2H), 0.88 (t, 3H, *J*=6.8). UPLC-MS (ESI): 310.3 ($M+H^+$).

S15: 1-(Piperidin-2-yl)undecan-1-ol

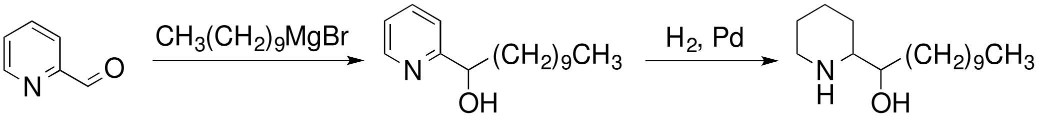

S15 precursor **S15**

Pyridine-2-carboxaldehyde (5.0 mL, 52 mmol) was added dropwise to a stirred solution of decylmagnesium bromide (52 mmol) in diethyl ether (52 mL) at 0 °C. The reaction mixture was allowed to reach room and after stirring at room temperature for an additional 4 h the reaction was quenched by addition of ice water. The mixture was extracted with diethyl ether, dried over $MgSO_4$, filtered, and concentrated under reduced pressure. The crude product was purified by flash chromatography on silica gel (ethyl acetate/hexanes 1:4), which afforded 1-(pyridin-2-yl)undecan-1-ol, the **S15** precursor, as an off-white solid (4.5 g, 35%). ^{1}H NMR ($CDCl_3$) δ: 8.52 (d, 1H, *J*=5.6), 7.65 (t, 1H, *J*=7.6), 7.23 (d, 1H, *J*=7.6), 7.17 (dd, 1H, *J*=7.6, 5.6), 4.71 (dd, 1H, *J*=7.6, 4.4), 4.13 (bs, 1H, -*OH*), 1.84–1.60 (m, 2H), 1.42–1.18 (m, 16H), 0.85 (t, 3H, *J*=7.0). UPLC-MS (ESI): 250.3 ($M+H^+$).

Hydrogenation according to the general procedure gave the product (**S15**) as a white solid (903 mg, 88%). ^{1}H NMR showed the product to be a 7:3 mixture of diastereomers.

Assessment of Anti-Proliferative Activity for Ceramide, Solenopsin A, and Analogs S11-S15

The affect of ceramide, solenopsin A, and analogs **S11-S15** was assessed on two normal cutaneous cell lines (primary melanocytes and primary keratinocytes), as well as on HaCaTs, which are immortalized hyperproliferative human keratinocytes. Primary melanocyte, primary keratinocyte, and HaCaT cells were plated at a concentration of 50,000 cells/well, 20,000 cells/well, and 15,000 cells/well respectively. The differences in starting cell concentration are not significant to the experiment. The cells were treated with 20 µM of ceramide C2, (-)-solenopsin A, (+)-solenopsin A, or analogs **S11-S15** for 24 hours, followed by cell counting with a Coulter Counter. All compounds were tested in quadruplicates. Of note, the primary keratinocytes were treated in serum free keratinocyte growth media. The primary melanocytes showed high toxicity to DMSO in serum-free media and were retested in complete melanocyte growth media. The HaCat cells were also tested in complete 10% DMEM media.

a.

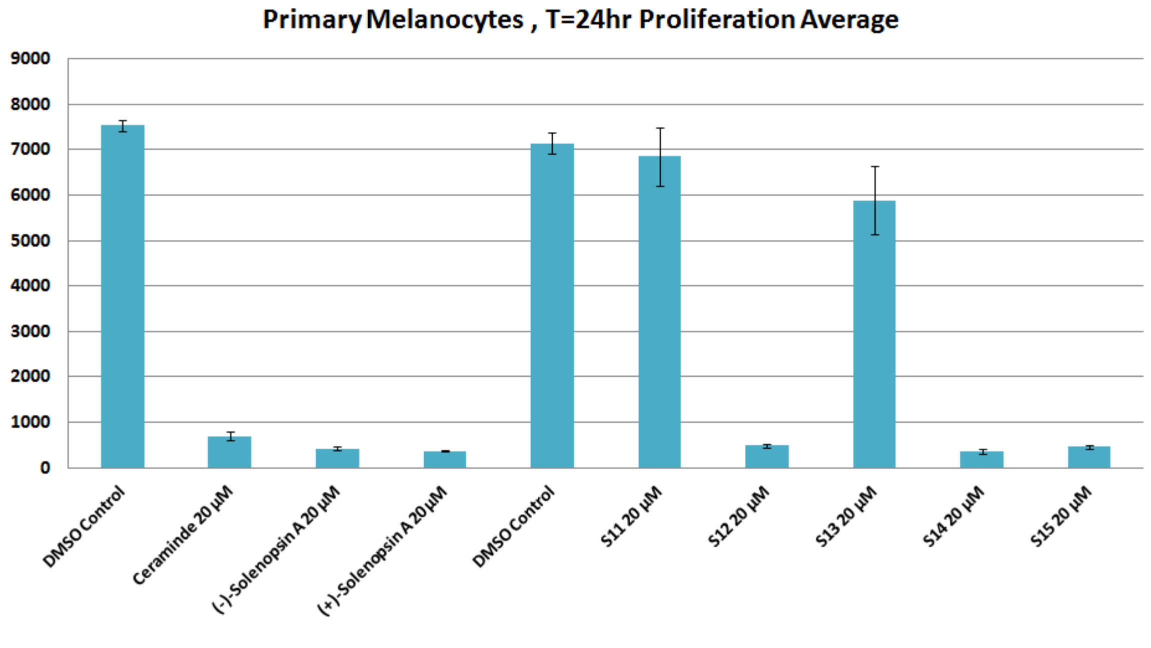

b.

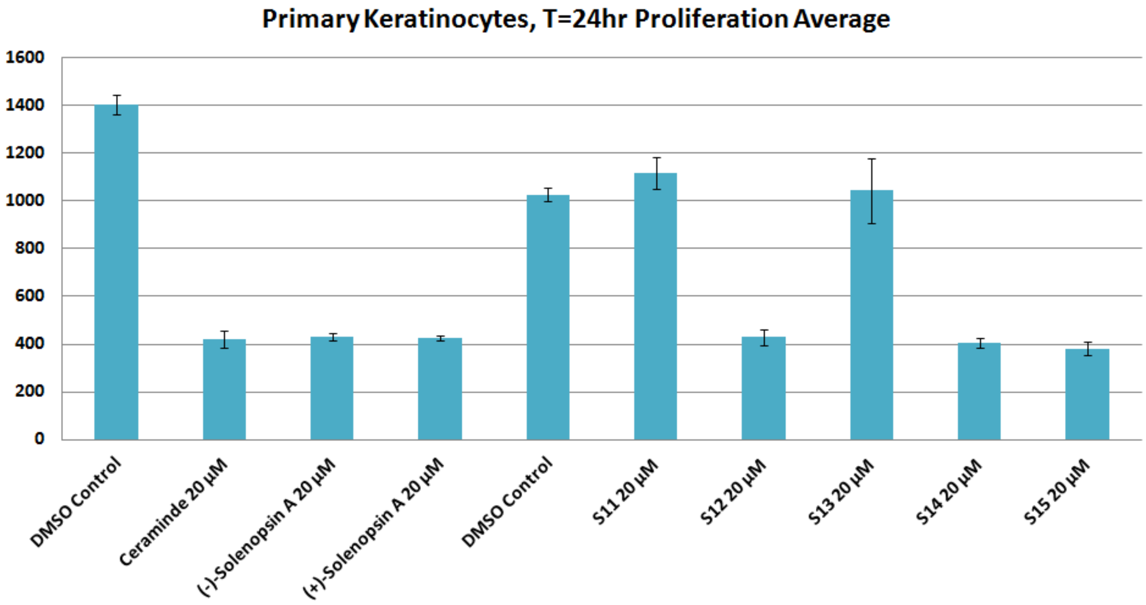

c.

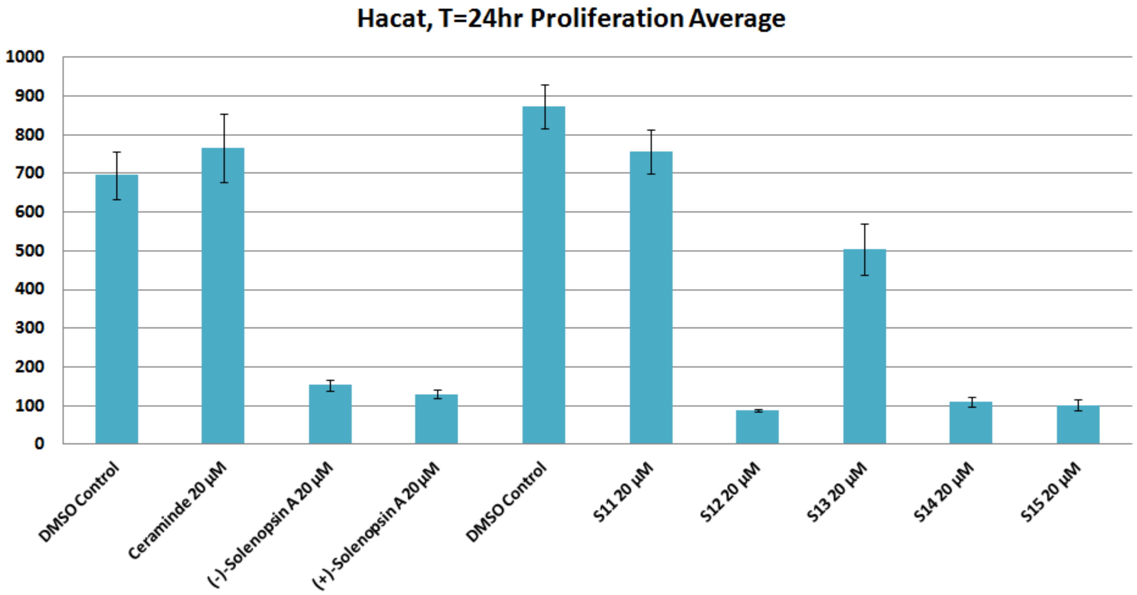

Figure S1 | Assessment of anti-proliferative activity for ceramide, solenopsin A, and analogs S11-S15.

The anti-proliferative effect of ceramide C2, (+)-solenopsin A, (-)-solenopsin A, and solenopsin analogs **S11**-**S15** were evaluated in (a) melanocyte cells, (b) primary keratinocyte cells, and (c) HaCaT cells. 50,000 cells/well (Melanocytes), 20,000 cells/well (Keratinocytes) 15,000 cells/well (HaCat) plated and treated for 24 h with each compound. The first DMSO bar in each chart serves as control for (+)-solenopsin A, (-)-solenopsin A, and ceramide C2. The second DMSO bar is the control for S11-S15. The displayed data are an average of four experiments ± s.d.

Akt activity and PDK1 activation - Cell Transfection and Imaging

Cell transfection and imaging was conducted as previously described (Gao *et al.*, 2011; Gao and Zhang, 2008). NIH 3T3 cells were plated and grown to 40% confluency. Cells were transfected with Lipofectamine 2000 and serum-starved for 24 hours and treated for 1 h with DMSO solutions of ceramide C2 (50 μM), (+)-solenopsin A (10 and 20 μM), (-)-solenopsin A (10 and 20 μM), and analogs **S11-S15** (10 μM). For imaging, cells were washed with Hank's balanced salt solution buffer once quickly, and then imaged in dark at room temperature in HBSS supplemented with solenopsin derivatives at indicated concentrations. Platelet derived growth factor (PDGF, 50 ng/mL) was added to assess the effects of the compounds on PDGF-induced Akt activity and PDK1 activation, Images were acquired on a Zeiss Axiovert 200M microscope with a cooled charge-coupled device camera. Dual-emission ratio imaging was performed with a 420DF20 excitation filter, a 450DRLP dichroic mirror, and two emission filters, 475DF40 and 535DF25 for CFP and YFP, respectively. The data was analyzed with Metafluor 6.2 software (Universal Imaging, Downingtown, PA). Cell regions were selected and fluorescence images were background-corrected by deducting the background (regions with no cells) from the emission intensities of CFP or YFP. FRET ratio of regions of interest (ROI) at cell cytosol and at cell periphery representing the plasma membrane were used, respectively. All the ratios were normalized with the ratio before PDGF addition.

Determination of Cytotoxic Capacity of Solenopsin A and Analogs S12 and S14

A 3D cell culture of human skin keratinocytes was used to determine any potential toxicity of Solenopsin A and the analogs **S12** and **S14**.

Chemicals. Psoriasis tissue model was obtained from MatTek™ Corporation (Ashland, MA), USA. Scott's reagents (Harleco), Xylene (Harleco), Eosin (Sigma Aldrich), Hematoxylin (Sigma Aldrich), Ethyl alcohol (Sigma Aldrich), and Cytoseal-60 (Richard-Allian) were used in H&E staining.

Sample preparation. Solenopsin A (mixture of (+) and (–) isomers) and analogs **S12** and **S14** and were dissolved in water and subsequent dilutions were done to attain a physiological concentration of 10µM.

Determination of cytotoxicity by MTT assay

MTT reagent was added to the tissue inserts after 72 hours treatment and incubated for 3 hours at 37°C with 5% CO2. After 3 hours, MTT was extracted from the tissues by extractant solution and placing the tissues on a shaker for two hours. Absorbance of extracted solution was measured at 570nm. Viable tissues will convert MTT to a purple dye and amount of conversion is proportional to the viability of the tissues. Cell viability was calculated using a spreadsheet provided by MatTek; viability of less than 50% was determined to be irritant and cytotoxic. The results in Figure S2 show that solenopsin A and analogs S12 and S14 were not toxic to the cells and resulted in cell viability of 59.0 ± 5.98%, 83.2.2 ± 12.81% and 83.1 ± 13.46%, suggesting that the dose delivered was not toxic or irritant to the tissues.

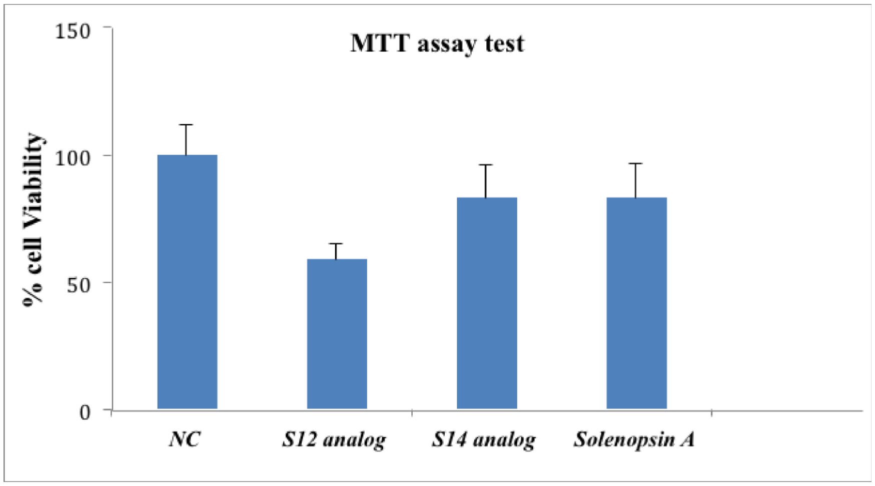

Figure S2 | Determination of cell viability and skin irritation by using MTT assay

MTT assay was performed on the psoriatic tissue model to determine cytotoxicity of dose given to the tissue. **(a)** NC- Negative control **(b)** S12 analog **(c)** S14 analog **(d)** Solenopsin A. Dosed tissue was treated with MTT reagent and then extracted followed by analysis by absorbance at 570nm. Cell viability for solenopsin and analogs; S12 and S14 were found to be more than 50%. Experiment was carried with n=4 replicates and all values represent mean ± standard deviation (p value < 0.05).

H&E staining.

Tissues from the culture inserts were taken and embedded in OCT media and stored at -80 degrees. 7µm thick tissues sections were obtained by cryotome and Polysine slides were used for the histology. Tissues samples were fixed using 10% formaldehyde. All tissues were stained using Hematoxylin and Eosin; subsequent washing was done using water, 95% alcohol, acid alcohol and scott's reagent. Slides were stored in Xylene until sealed with cytostat-60. Acid alcohol solution was prepared by adding 2ml of HCL in 198ml of 70% EtOH. Images were viewed using Leica polarizing microscope and image analysis was done using ImageJ software.

Translocation of PTEN to Membrane Rafts

We noted the structural similarity between solenopsin and ceramide and therefore wanted to investigate if solenopsin also recruits PTEN to membrane rafts. To do this we used sucrose density gradient fractionation followed by western blotting. Caveolin was used as a positive marker to identify lipid raft fractions and an anti-PTEN antibody was used to identify PTEN-containing fractions (Figure S1). A375 cells were treated for 1 h with 20 μM of (+)-solenopsin, (-)-solenopsin, or analogs **S12-S15**. Cells treated with only DMSO were used as a negative control and cells treated with 50 μM of ceramide were used as positive control. As expected, ceramide treated cells display a higher amount of PTEN in the lipid raft fractions (fraction 1-4) (Figure S1). The compound with the largest amount of PTEN in the raft fractions was (-)-solenopsin A (Figure S1). (+)-Solenopsin A and analog **S12**-treated cells showed similar amounts of PTEN in the raft fractions as the ceramide treated cells (Figure S1). The rest of the analogs (**S13-S15**) appeared to have similar or even lower amounts of PTEN in lipid rafts as the control group (Figure S1).

Sucrose Density Gradient Fractionation

Cells were grown in T-75 flasks until 80% confluent, followed by treatment for 1 h with 20 μM DMSO solutions of (+)-solenopsin A, (-)-solenopsin A, analogs (**S12-S15**), or 50 μM of ceramide C2. Lipid raft fractionation was performed with a 5-40% sucrose discontinuous gradient as previously described (Chen *et al.*, 2011; Huang *et al.*, 2010). The A375 cells from each treatment group were subjected to mechanical disruption with 8 strokes of a homogenizer and lysed for 30 min on ice in 650 μL 0.5% Brij96 in TNEV buffer (10 mM Tris·HCl, pH 7.5, 150 mM NaCl, 5 mM EDTA, 2 mM Na vanadate, and protease inhibitor cocktail). The homogenates were centrifuged at 1000 rpm for 1 min at 4 °C. Supernatant (500 μL) was diluted 1:1 with 80% sucrose in TNEV buffer and transferred to a centrifuge tube (13 × 51 mm). Next, a layer of 35% sucrose in TNEV buffer (3 mL) was carefully placed on top of the first layer, followed by a 5% sucrose layer in TNEV buffer (1 mL). The sucrose gradient tube was centrifuged at 34,000 rpm for 22 h at 4 °C in a Beckman SW 50.1 rotor.

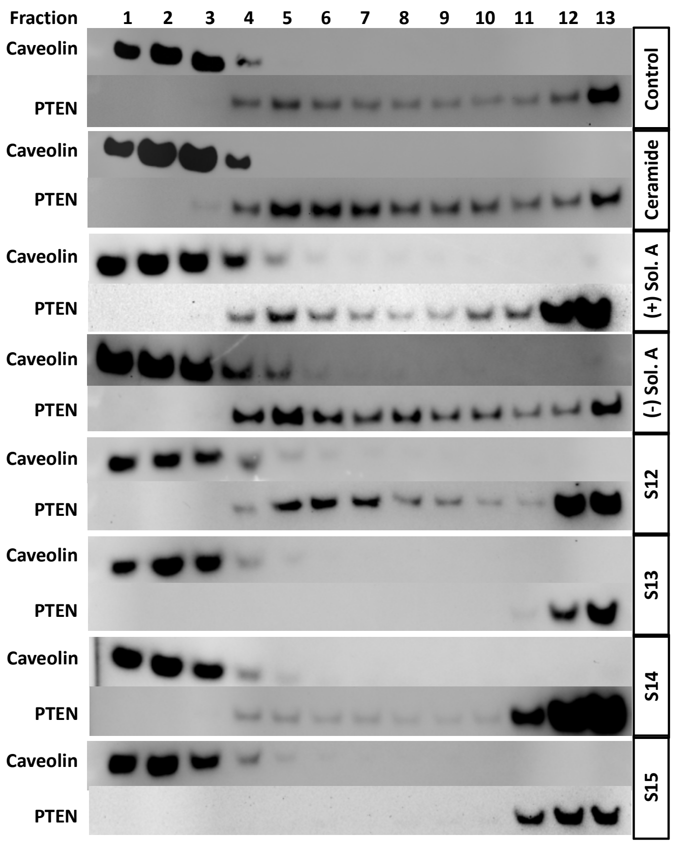

Figure S3 | Lipid raft fractionation

A375 cells were treated for 1 h with: ceramide C2 (50 µM), (+)-solenopsin A ((+) Sol. A, 20 µM), (-)-solenopsin A ((-) Sol. A, 20 µM), or solenopsin analogs **S12**-**S15** (20 µM). Cells were lysed in 0.5% Brij96 in TNEV, loaded onto the 5-40% sucrose gradient, and centrifuged at 34,000 rpm at 4 °C for 22 h. Thirteen fractions were collected, starting from the top of the tube. Equal volumes of each fraction were analyzed by Western blotting with caveolin and PTEN antibodies. Caveolin serves a positive control for which fractions that contain lipid rafts.

Solenopsin A and Analogs Effect on Signaling Pathways

A375 (human melanoma), SVR (murine angiosarcoma), and A2058 (human melanoma) cells treated with solenopsin A and analogs were evaluated by Western-blotting with p-Akt S473, p-MAPK 44/42, and B-actin (Figure S2). The Western blots that can be seen in Figure S2 were quantified by densitometry analysis using Image Lab (version 4.0, build 16).

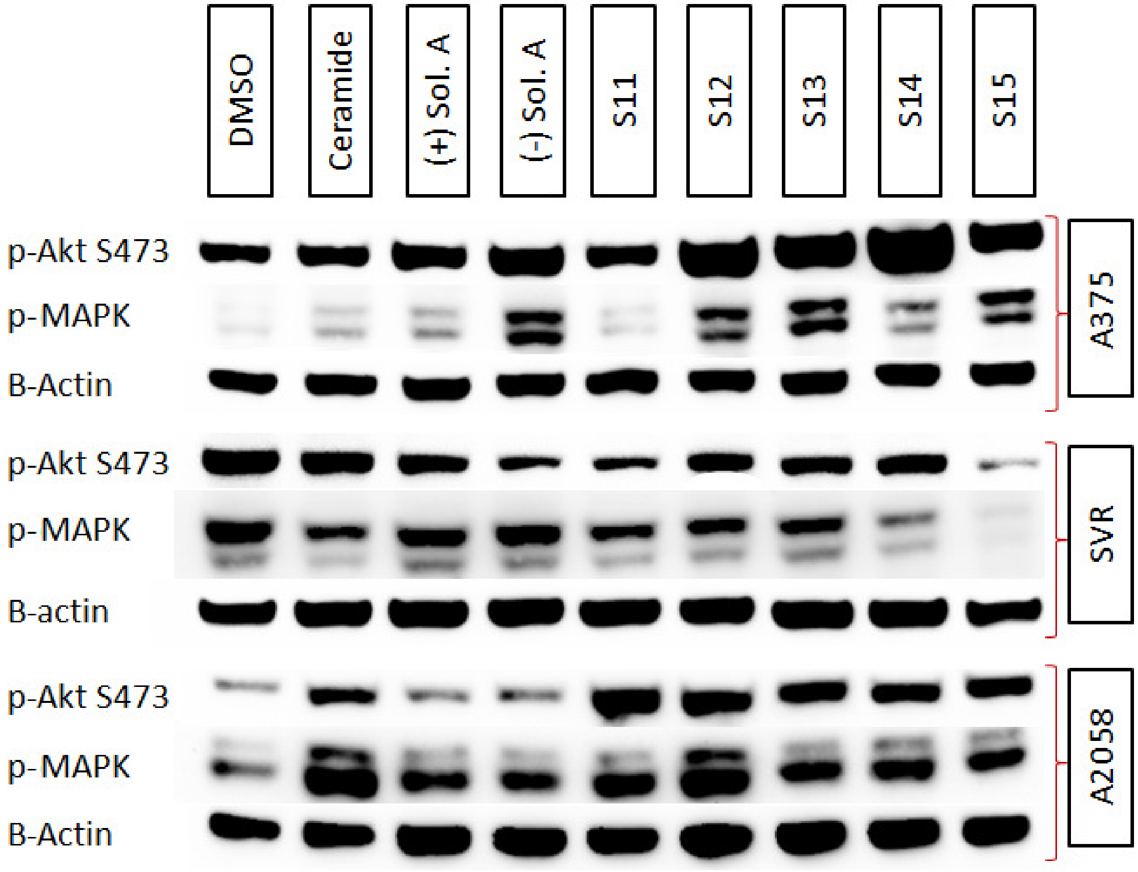

Figure S4 | The effect of solenopsin A and analogs on the expression of p-Akt and p-pMAPK 44/42.

A375, SVR, and A2058 cells were treated for 24 h with ceramide C2 (20 µM), (+)-solenopsin A ((+) Sol. A, 10 µM), (-)-solenopsin A ((-) Sol. A, 10 µM), or solenopsin analogs S11-S15 (10 µM). The expression of p-Akt S473, p-MAPK 44/42, and B-actin was determined by Western blotting.

Table S1 | Quantification of Western Blots

a. A375 cells

	A375 p-AKT S473	A375 p-Mapk 44	A375 p-Mapk 42	A375 B-actin
	Rel. Quant.	Rel. Quant.	Rel. Quant.	Rel. Quant.
DMSO	1	1	1	1
Ceramide	1.208054	2.141506	1.862485	1.140405
(+) Sol. A	1.612894	3.940761	1.988622	1.137794
(-) Sol. A	2.128378	6.932176	3.487396	1.243729
S11	1.192409	2.374528	1.483867	1.280489
S12	2.968518	5.423433	2.4409	1.192454
S13	2.914597	7.016663	3.833042	1.208999
S14	4.226653	4.475294	2.122226	1.150122
S15	2.103898	7.212949	2.813118	1.159501

b. SVR cells

	SVR p-AKT S473	SVR p-Mapk 44	SVR p-Mapk 42	SVR B-actin
	Rel. Quant.	Rel. Quant.	Rel. Quant.	Rel. Quant.
DMSO	1	1	1	1
Ceramide	0.808427	0.635507	0.546842	1.328731
(+) Sol. A	0.576818	0.886079	1.239762	1.57345
(-) Sol. A	0.332639	0.848158	0.891279	1.572554
S11	0.338824	0.668133	0.673245	1.509688
S12	0.476873	0.655229	0.774494	1.44067
S13	0.518773	0.721647	1.005254	1.689692
S14	0.639281	0.402463	0.629078	1.448407
S15	0.200993	0.101492	0.056383	0.891902

c. A2058 cells

	A2058 p-AKT S473	A2058 p-Mapk 44	A2058 p-Mapk 42	A2058 B-actin
	Rel. Quant.	Rel. Quant.	Rel. Quant.	Rel. Quant.
DMSO	1	1	1	1
Ceramide	2.501157	4.958665	4.958665	1.198429
(+) Sol. A	1.327881	2.010908	2.010908	2.500594
(-) Sol. A	1.381013	1.79967	1.79967	2.012956
S11	4.10237	2.422732	2.422732	1.391788
S12	3.026043	4.008375	4.008375	1.962398
S13	2.81174	1.782164	1.782164	2.384119
S14	2.944344	2.13083	2.13083	2.019733
S15	2.826213	1.567815	1.567815	2.28566

Autophagosome Staining to study Mitochondrial Function

UM-SCC1A cells were treated with DMSO (control) and 10 µM of (-)-solenopsin A or analog S14 for 18 h. The cells were stained using a Cyto-ID Autophagy Staining assay from Life Technologies.

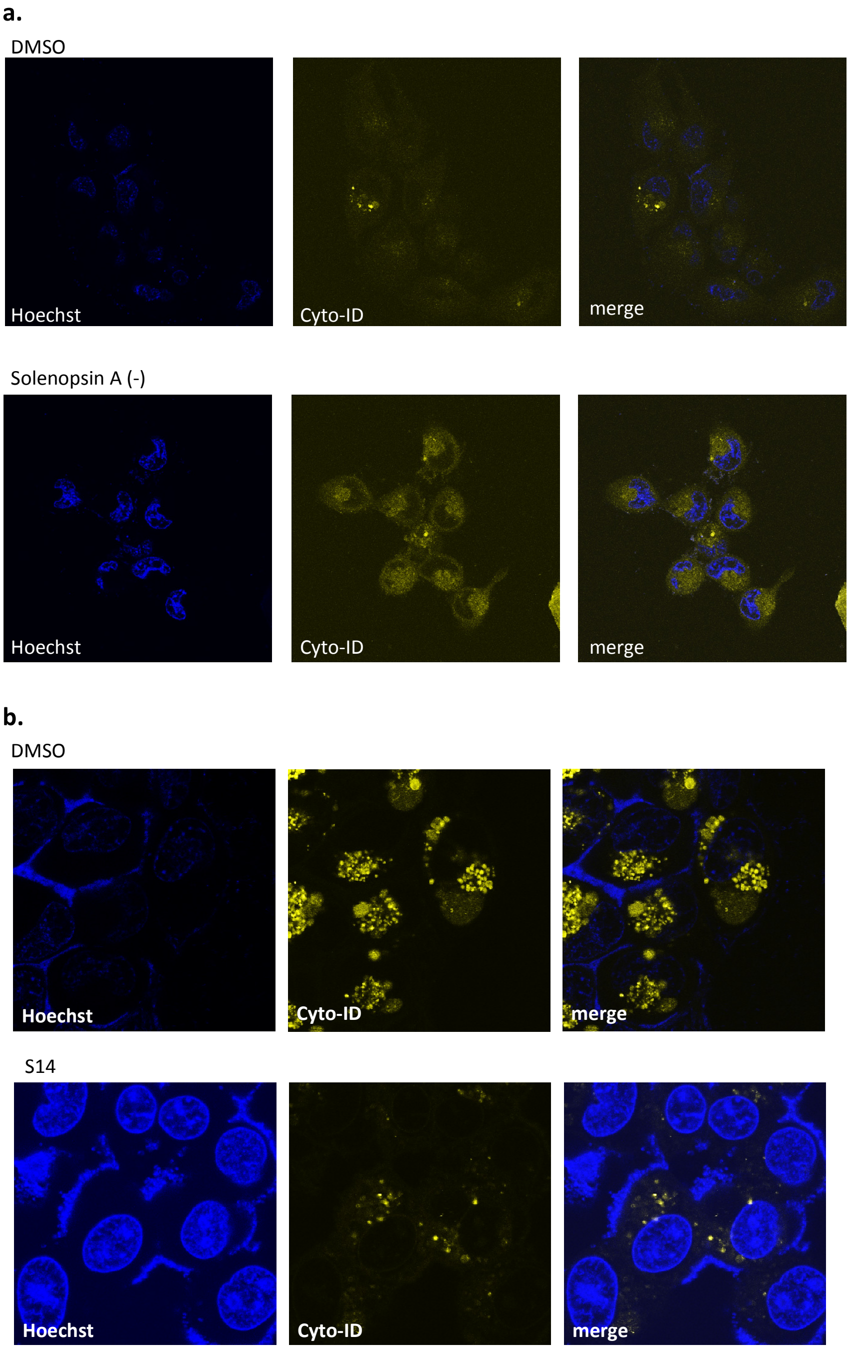

Figure S5 | Autophagy staining confirms that solenopsin A induces autophagy

A Cyto-ID Autophagy Staining assay from Life Technologies was used to investigate if (-) solenopsin A and analog **S14** induce autophagy. The dye stains autophagosomes and co-localizes with LC3-II. Increased fluorescence is an indication of autophagy. UM-SCC1A cells were treated with (**a**) DMSO (control) and 10uM of (-) solenopsin A or (**b**) DMSO (control) and analog S14 for 18 h.

Measurement of ROS with dihydroethidium (DHE)

A375 and SVR cells were plated in 6 cm dishes. After cells had adhered to the plates, they were treated for 24 h with 10 μM of (+)-solenopsin A, (-)-solenopsin A, or analogs (S11-S15) in DMSO at which time the media was aspirated. Cells were washed with 2 mL PBS then treated with 0.05 % trypsin/0.53 mM EDTA for 3 min at RT. Cells were collected and then pelleted at 600g for 2 min. The supernatant was aspirated and the cells were suspended in 10 μM DHE and incubated for 10 min in the dark at RT while shaking. Following this incubation, cells were kept until counting on a Becton Dickinson FACScan flow cytometer. 10,000 cells were counted and analyzed by FlowJo 7.6.4. Mean values of DHE fluorescence intensity were compared and all samples were repeated in triplicate.

References

Chen G, Howe AG, Xu G*, et al.* (2011) Mature N-linked glycans facilitate UT-A1 urea transporter lipid raft compartmentalization. *FASEB journal : official publication of the Federation of American Societies for Experimental Biology* 25:4531-9.

Gao X, Lowry PR, Zhou X*, et al.* (2011) PI3K/Akt signaling requires spatial compartmentalization in plasma membrane microdomains. *Proceedings of the National Academy of Sciences of the United States of America* 108:14509-14.

Gao X, Zhang J (2008) Spatiotemporal analysis of differential Akt regulation in plasma membrane microdomains. *Molecular biology of the cell* 19:4366-73.

H. M. T. Bandara Herath, Nanayakkara NPD (2008) Synthesis of enantiomerically pure fire ant venom alkaloids: Solenopsins and isosolenopsins A, B and C. *J Heterocyclic Chem* 45:129-36.

Huang H, Feng X, Zhuang J*, et al.* (2010) Internalization of UT-A1 urea transporter is dynamin dependent and mediated by both caveolae- and clathrin-coated pit pathways. *American journal of physiology Renal physiology* 299:F1389-95.

Pianaro A, Fox EGP, Bueno OC*, et al.* (2012) Rapid configuration analysis of the solenopsins. *Tetrahedron-Asymmetr* 23:635-42.
